# Supplementary material for: Attempts to Understand Oral Mucositis in Head and Neck Cancer Patients through Omics Studies: A Narrative Review
Source: Int J Mol Sci. 2023 Nov 30;24(23):16995. doi: 10.3390/ijms242316995 (PMC10706892; doi:10.3390/ijms242316995)

Supplementary Table 1. Search strategy for selection of literature.

Database(s): Ovid MEDLINE(R) ALL 1946 to October 27, 2022
Search Strategy:

#	Searches	Results
1	exp Mucositis/	1887
2	exp Stomatitis/	18189
3	(mucositides or mucositis or stomatitides or stomatitis).ti,ab,kf.	27288
4	1 or 2 or 3	38889
5	exp Genetics/	306013
6	exp Biomarkers/	862602
7	exp Genomics/	145470
8	(Genetic* or genomic* or biomarker* or genome or gene or transcription).ti,kf.	1330928
9	ge.fs.	3878104
10	exp Genetic Predisposition to Disease/	155031
11	exp Whole Genome Sequencing/	16404
12	exp High-Throughput Nucleotide Sequencing/	49827
13	exp transcription, genetic/	234621
14	exp Gene Expression Profiling/	156072
15	exp Gene Expression/	492942
16	exp Genetic Variation/	1176355
17	Single Nucleotide Polymorphism.ti,ab,kf.	35285
18	(Proteomics* or proteomics or proteome or transcriptome or epigenomic* or transcriptomic*).ti,ab,kf.	204417
19	exp Metabolomics/	25218
20	(Metabolomic or Metabolomics or metabolome or metabolit*).ti,ab,kf.	342518
21	exp Microbiota/	67133
22	(microbiomics or microbiome or microbiomes or microbiota or microbiotas or microflora or microorganism* or "micro organism*" or microbiological or microbial or flora or microbes or microbium).ti,ab,kf.	483503

23	mi.fs.	825251
24	exp *MICROBIOLOGY/	95303
25	or/5-24	6266612
26	4 and 25	9161
27	exp *Mucositis/ or exp *Stomatitis/	14605
28	(stomatitis or mucositis).ti,kf.	10293
29	27 or 28	19186
30	26 and 29	4359
31	limit 30 to (english language and yr="2003 -Current")	2484
32	exp Neoplasms/	3750717
33	(cancer* or carcinom* or tumor* or tumour* or neoplas* or malignan* or metasta* or myeloma* or leuk?emia* or lymphoma* or sarcoma* or melanoma* or radiotherapy or radiation or chemotherapy).ti,ab,kf.	4534839
34	32 or 33	5270436
35	31 and 34	766
36	case reports.pt. not (exp clinical study/ or comparative study/ or evaluation studies/ or meta-analysis/ or multicenter study/ or validation studies/ or letter.pt.)	2049846
37	case report*.ti,jn.	333747
38	case reports.pt. not (((case adj4 series) or cohort or retrospective*).ti,ab. or exp Cohort Studies/)	2171662
39	36 or 37 or 38	2348630
40	35 not 39	728
41	((intestinal or gastrointestinal) not oral).ti.	197358
42	40 not 41	592
43	(animals not (humans and animals)).sh.	5025007
44	(mice or mouse or murine or rat or rats or rodent or cells or "in vitro" or "cell line").ti.	2703628
45	43 or 44	6158589
46	42 and 45	167
47	42 not 46	425

Supplementary Figure 1: Search Strategy and PRISMA Flow Diagram for literature review based on Page et al. [76]. For more information on PRISMA, visit http://www.prisma-statement.org/.

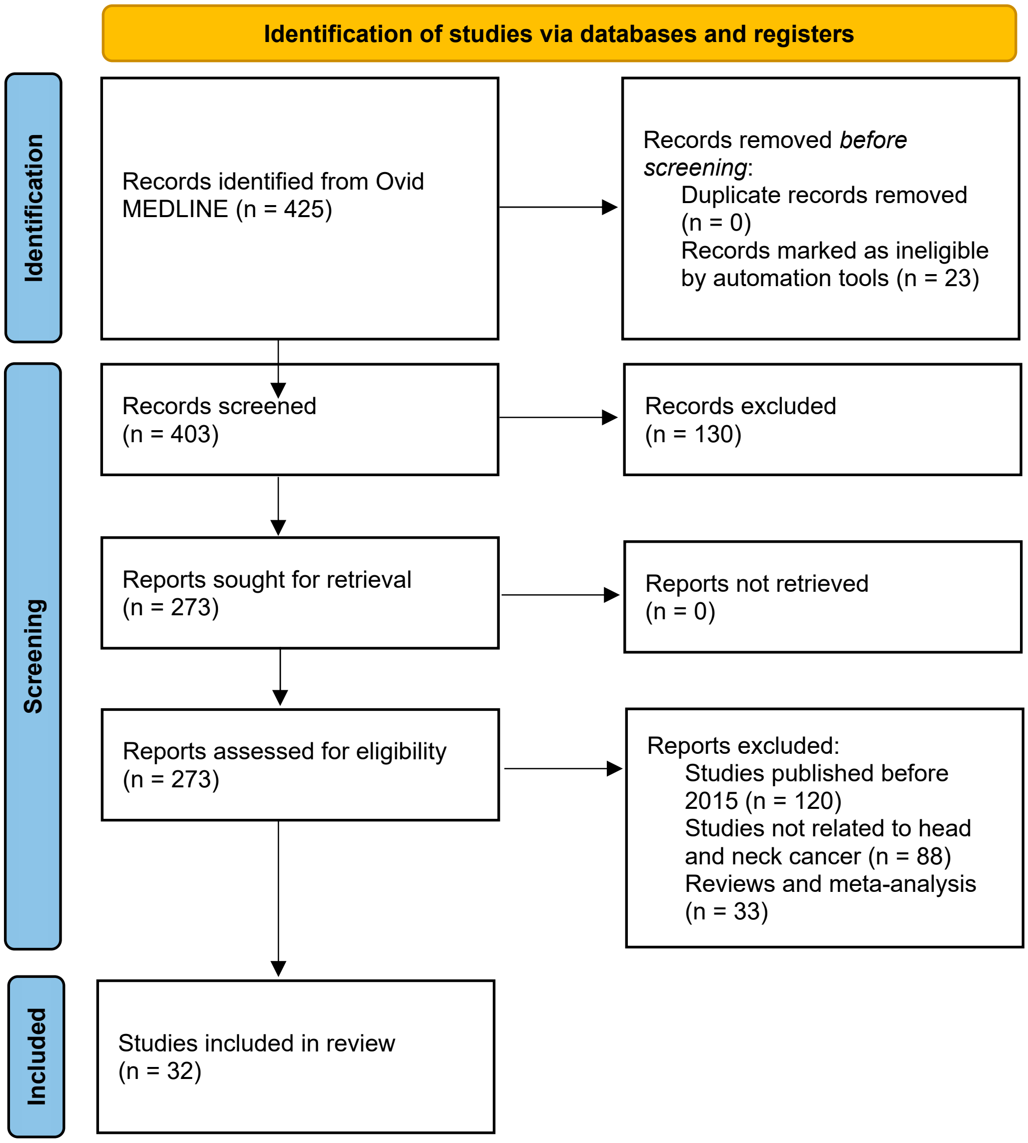

Supplement: Supplementary file 1 [file ijms-24-16995-s001.zip › ijms-2732027-supplementary.pdf]
